# Unravelling the obesity epidemic among young people in Qatar: a literature review

**DOI:** 10.3389/fpubh.2026.1887866

**Published:** 2026-08-17

**Authors:** Mohammed Ali Abdulla, Jassim Khalid Al-Hail, Christine Gaskell

**Affiliations:** Weill Cornell Medicine - Qatar, Doha, Qatar

**Keywords:** children and adolescence, diet, nutrition, obesity, public health, Qatar, ultra-processed foods

## Abstract

**Background:**

The prevalence of childhood and adolescent obesity has risen dramatically over recent decades, particularly in rapidly developing countries undergoing significant dietary transition. Qatar now has one of the highest rates of childhood overweight and obesity globally, coinciding with increased availability and consumption of processed and ultra-processed foods (UPFs). Although associations between UPF consumption and obesity are well established, the biological and behavioural mechanisms underpinning this relationship have not previously been synthesised within the context of Qatar.

**Objective:**

To provide a structured narrative review of the evidence describing the mechanisms through which processed and ultra-processed foods contribute to obesity among children and adolescents, while contextualising these findings within Qatar’s changing food environment.

**Findings:**

Evidence consistently demonstrates that processed and ultra-processed foods promote excessive energy intake through five interconnected mechanisms: high energy density and increasing portion sizes; impaired appetite and satiety regulation; displacement of nutrient-dense whole foods; activation of reward pathways that encourage compulsive eating behaviours; and neurobiological and metabolic adaptations that may reinforce long-term overconsumption. Although much of the available evidence originates from Western populations, similar dietary transitions are occurring across Qatar and the wider Gulf region, suggesting these mechanisms are highly relevant to the local context. However, important limitations remain, including the relative scarcity of longitudinal and intervention studies conducted within Middle Eastern populations.

**Conclusion:**

Addressing childhood obesity in Qatar requires interventions that extend beyond calorie reduction to target the broader food environment and dietary quality. Strengthening school nutrition policies, promoting traditional dietary practices, improving nutrition education, and reducing children’s exposure to ultra-processed food marketing should form key components of future public health strategies. Further longitudinal research conducted within Qatar is required to better understand the long-term health consequences of increasing UPF consumption among young people.

## 1 Introduction

Childhood obesity has become one of the most significant global public health challenges of the twenty-first century. According to the World Health Organization, the prevalence of overweight and obesity among children and adolescents has increased substantially over recent decades, contributing to an increased burden of type 2 diabetes, cardiovascular disease, metabolic dysfunction, certain cancers, and poorer psychological wellbeing later in life. Although obesity is recognised as a multifactorial disease influenced by genetic, behavioural, environmental, and socioeconomic determinants, dietary change remains one of the principal modifiable risk factors (1, 2).

Among dietary factors, increasing consumption of processed and ultra-processed foods (UPFs) has emerged as an important contributor to excessive weight gain. UPFs are industrial formulations manufactured predominantly from refined food substances and additives, often containing little or no intact whole foods. These products are typically characterised by high energy density, elevated levels of added sugars, saturated fats and sodium, together with reduced fibre, protein, vitamins and minerals (3, 4). Their formulations are specifically designed to maximise palatability, convenience, shelf life, and commercial appeal, making them particularly attractive to children and adolescents.

Processed and fast foods comprise a wide range of industrially manufactured products, spanning from moderately processed items with limited ingredient modification to ultra-processed foods (UPFs) containing multiple industrial additives and artificial components. The NOVA food classification system categorises foods according to the nature, extent and purpose of industrial processing into four groups: (i) unprocessed or minimally processed foods, (ii) processed culinary ingredients, (iii) processed foods and (iv) ultra-processed foods. Unlike nutrient-based classification systems, NOVA considers the degree of industrial processing as the defining characteristic of foods, providing a framework for investigating associations between food processing, dietary quality and health outcomes (3). This review focuses primarily on processed foods (Group 3) and ultra-processed foods (Group 4), with particular attention to fast foods, which are predominantly classified as ultra-processed foods because of their formulation and manufacturing processes.

Fast foods, including burgers, fried chicken, pizzas, sugar-sweetened beverages, confectionery, and packaged snacks, represent some of the most commonly consumed UPFs among young people (5). These foods combine high energy density with low nutritional value and are frequently marketed through sophisticated advertising campaigns targeting children and adolescents (6). Their widespread availability, affordability, and convenience have substantially altered dietary habits worldwide.

### 1.1 Nutrition transition in Qatar

The rapid economic development experienced by Qatar over the past five decades has transformed almost every aspect of daily life, including food production, food availability, and dietary behaviours. Historically, traditional Qatari diets were centred on minimally processed foods including fish, seafood, whole grains, dates, legumes, fermented dairy products, and modest amounts of locally produced meat (7, 8). Meals were predominantly prepared at home and reflected cultural practices that emphasised moderation and shared family eating.

However, economic prosperity, urbanisation, globalisation, and increased reliance on imported foods have driven a marked nutrition transition (7, 8). Traditional dietary patterns have increasingly been replaced by Westernised diets characterised by greater consumption of fast foods, sugar-sweetened beverages, packaged snacks, and ready-to-eat meals. The rapid expansion of international restaurant chains, online food delivery platforms, and convenience foods has further accelerated this transition, particularly among younger generations (9).

These dietary changes have occurred alongside broader lifestyle modifications including reduced physical activity, increased sedentary behaviour, greater screen time, and increased reliance on motorised transport (7). Together, these environmental changes have created an obesogenic environment that promotes positive energy balance and excessive weight gain. Consequently, Qatar now reports one of the highest prevalences of childhood overweight and obesity in the Gulf region, with recent estimates indicating that approximately 27.7% of children aged 5–14 years are living with obesity (10).

Although obesity results from chronic positive energy balance, increasing evidence suggests that processed and ultra-processed foods influence body weight through mechanisms extending beyond calorie content alone. Their physical structure, nutrient composition, and sensory characteristics may alter appetite regulation, eating behaviour, metabolic responses, and brain reward pathways, thereby encouraging habitual overconsumption (3, 11, 12). These mechanisms operate synergistically and may reinforce one another over time, particularly during childhood and adolescence when dietary preferences and eating behaviours are still developing.

Recent systematic reviews and controlled feeding studies have strengthened evidence linking UPFs with obesity and metabolic disease. Randomised dietary intervention studies demonstrate that diets rich in UPFs increase daily energy intake and promote rapid weight gain even when macronutrient composition is matched with minimally processed diets (3, 11). Large prospective cohort studies similarly report positive associations between UPF consumption and obesity (12, 13).

Importantly, the current evidence base is heterogeneous. While associations between UPF consumption and obesity are consistently reported, differences in study design, dietary assessment methods, definitions of food processing, and participant characteristics complicate direct comparison across studies (12, 13). Furthermore, much of the available literature remains observational, limiting causal inference. Therefore, critical evaluation of the quality and limitations of existing evidence is essential when interpreting the role of processed foods in the obesity epidemic.

### 1.2 Aim of this review

The aim of this structured narrative review is to critically synthesise current evidence describing the biological and behavioural mechanisms through which processed and ultra-processed foods contribute to obesity among children and adolescents, with particular emphasis on their relevance to Qatar. Specifically, this review examines five interconnected mechanisms:

1. High energy density and increasing portion sizes.
2. Disruption of appetite and satiety regulation.
3. Nutritional displacement of whole, nutrient-dense foods.
4. Reward-driven and addictive eating behaviours; and
5. Neurobiological and metabolic adaptations associated with habitual UPF consumption.

In addition to summarising current evidence, this review critically evaluates the strengths and limitations of the existing literature, identifies gaps relevant to Qatar and the wider GCC region, and discusses implications for future research, clinical practice, nutrition education, and public health policy.

## 2 Methods

This review was conducted as a structured narrative review to synthesise current evidence examining the relationship between processed and ultra-processed food (UPF) consumption and obesity among children and adolescents, with particular emphasis on the relevance of these findings to Qatar. A structured narrative approach was selected because the available literature comprises a diverse range of study designs, including randomised controlled trials, prospective cohort studies, cross-sectional studies, systematic reviews, and national policy reports, making quantitative synthesis inappropriate.

### 2.1 Literature search strategy

A comprehensive literature search was undertaken using the electronic databases PubMed, Scopus, and Google Scholar. These databases were selected because they collectively provide broad coverage of biomedical, nutrition, epidemiological, and public health research. Searches were conducted for articles published between January 2000 and March 2026, reflecting the period during which consumption of processed and ultra-processed foods has become increasingly recognised as a contributor to obesity.

The following search strategy was used with appropriate modifications for each database:

(“Obesity” OR “Overweight”) AND (“Qatar” OR “Middle East” OR “Gulf Cooperation Council” OR “GCC”) AND (“Nutrition” OR “Diet”) AND (“Processed food” OR “Ultra-processed food” OR “UPFs” OR “Fast food”) AND (“Children” OR “Adolescents”) AND (“Public health” OR “Policy”).

Medical Subject Headings (MeSH), where available, were combined with free-text keywords using Boolean operators (AND/OR) to maximise retrieval of relevant literature. Reference lists of eligible studies and relevant review articles were also manually screened to identify additional publications not captured during the electronic database search.

### 2.2 Eligibility criteria

Studies were included if they:

- Investigated human participants;
- Examined processed or ultra-processed food consumption and obesity or obesity-related outcomes;
- Evaluated dietary patterns, nutrition, eating behaviours, or biological mechanisms associated with obesity;
- Included children, adolescents, or young adults, or provided evidence considered relevant to these age groups;
- Were conducted in qatar, other gulf cooperation council (gcc) countries, or internationally where findings were applicable to the objectives of this review;
- Were published as peer-reviewed original research articles, systematic reviews, meta-analyses, randomised controlled trials, prospective cohort studies, or national public health reports (6).

Studies published in adult populations were also considered where they provided mechanistic evidence relating to appetite regulation, metabolic responses, or neurobiological pathways that are applicable across age groups.

Studies were excluded if they:

- Focused on dietary exposures unrelated to processed or ultra-processed foods;
- Did not examine obesity or obesity-related outcomes;
- Consisted solely of conference abstracts, editorials, opinion articles, or commentaries;
- Were not available in English.

### 2.3 Study selection

The database searches initially identified a broad body of potentially relevant literature. Titles and abstracts were screened for relevance to the review objectives, after which full-text articles were assessed against the predefined eligibility criteria. Studies judged to provide the highest level of evidence, including systematic reviews, meta-analyses, randomised controlled trials, and prospective cohort studies, were prioritised. Additional observational studies were included where they contributed important mechanistic or regional evidence, particularly relating to Qatar or the wider GCC region.

As this review was narrative rather than systematic, a formal PRISMA flow diagram was not generated. Nevertheless, a structured and transparent screening process was followed to minimise selection bias and ensure that included studies were directly relevant to the review objectives.

### 2.4 Data synthesis

Following study selection, findings were synthesised narratively rather than quantitatively because of substantial heterogeneity in study design, participant characteristics, dietary assessment methods, definitions of processed food exposure, and reported outcome measures.

The studies were organized into five interconnected biological and behavioural mechanisms through which processed and ultra-processed foods may contribute to obesity, as mentioned in section 1.2.

This thematic approach enabled integration of evidence from experimental studies, epidemiological investigations, and public health literature while maintaining a coherent biological framework relevant to childhood obesity in Qatar.

### 2.5 Critical appraisal of the evidence

Although a formal risk-of-bias assessment was not undertaken because of the narrative design of the review, the strength of the available evidence was considered throughout the synthesis. Greater emphasis was placed on findings from systematic reviews, meta-analyses, randomised controlled trials, and large prospective cohort studies, while evidence derived from cross-sectional and observational studies was interpreted more cautiously owing to their inability to establish causality (Table 1).

**Table 1:** Summary of key studies investigating processed and ultra-processed foods and obesity.

Study	Country/population	Study design	Main findings	Level of evidence
Marino et al. (13)	International	Systematic Review	Higher UPF consumption associated with increased obesity prevalence globally.	High
Gupta et al. (4)	USA	Nutrient Profile Analysis	UPFs contained approximately twice the energy density of minimally processed foods and lower nutrient density.	Moderate
Hall et al. (11)	USA	Randomised Controlled Feeding Trial	UPF diet increased daily energy intake by approximately 500 kcal/day and resulted in significant weight gain.	High
Hamano et al. (14)	Japan	Randomised Crossover Trial	Participants consuming UPFs gained significantly more body fat and consumed meals more rapidly.	High
Papagiannaki and Kerr (15)	International	Narrative Review	Increasing portion sizes contribute to obesogenic food environments.	Moderate
Hollands et al. (22)	International	Cochrane Review	Larger portions, packaging and tableware increase food consumption.	High
Flood-Obbagy and Rolls (18)	USA	Experimental Study	Whole foods produced greater satiety than processed forms despite similar energy content.	Moderate
Morys et al. (43)	International	Neuroimaging Study	Higher UPF intake associated with altered brain structure independent of adiposity.	Moderate

Level of evidence was assigned based on study design. High was assigned to systematic reviews, meta-analyses, Cochrane reviews, and randomised controlled trials. Moderate was assigned to narrative reviews, controlled experimental studies, and cross-sectional studies.

Particular attention was also given to study limitations, including short intervention durations, reliance on self-reported dietary assessment, heterogeneous definitions of ultra-processed foods, and the limited availability of studies conducted within Qatar and other Middle Eastern populations. These considerations informed the critical discussion presented throughout the review and the identification of future research priorities.

### 2.6 Relevance to the Qatari context

Evidence from international populations was interpreted within the context of the country’s rapid nutrition transition, changing food environment, and increasing prevalence of childhood obesity. Where available, findings from Qatar and neighbouring GCC countries were prioritised to improve the regional relevance of the review. This approach enabled the integration of robust international evidence while acknowledging important cultural, environmental, and socioeconomic differences that may influence dietary behaviours and obesity risk in the Qatari population.

## 3 Energy density characteristics of processed foods

Energy density, defined as the amount of energy (kcal) per gram of food, is recognised as one of the principal characteristics through which processed and ultra-processed foods (UPFs) promote excessive energy intake and obesity (2). Compared with minimally processed foods, UPFs typically contain greater quantities of refined carbohydrates, added sugars, saturated fats, and industrial ingredients while containing substantially less dietary fibre and water. This combination increases caloric density without proportionally increasing satiety, encouraging passive overconsumption.

A systematic review by Marino et al. examining worldwide UPF consumption reported that UPFs constitute a substantially greater proportion of the diets of overweight and obese individuals than of healthy-weight populations (13). Although the review included studies from multiple countries and age groups, considerable heterogeneity existed in dietary assessment methods and definitions of UPFs. Nevertheless, the consistency of findings across diverse populations strengthens the evidence linking higher UPF consumption with increased obesity risk.

The nutritional characteristics of UPFs further support this association. Gupta et al. demonstrated that UPFs contain approximately twice the energy density of minimally processed foods while providing considerably lower nutrient density (4). Foods with high energy density permit greater caloric intake before physiological satiety signals are activated, thereby increasing total daily energy consumption (2, 4).

One of the strongest pieces of evidence supporting a causal relationship between UPF consumption and weight gain comes from controlled dietary intervention studies. Hall et al. conducted a randomised inpatient crossover trial in which participants consumed either an ultra-processed or minimally processed diet matched for total calories, macronutrients, sugar, sodium and fibre (11). Despite the nutritional matching, participants consuming the UPF diet consumed approximately 500 kcal/day more and experienced significant increases in body weight over only two weeks (11). This landmark study demonstrated that characteristics intrinsic to UPFs, beyond nutrient composition alone, may influence eating behaviour and energy intake.

Similarly, Hamano et al. reported that participants consuming a one-week UPF diet gained more than 1 kg of fat compared with participants consuming minimally processed foods with similar macronutrient profiles (14). Importantly, participants consuming UPFs ate more rapidly, suggesting that food texture and oral processing contribute to increased caloric intake. These findings support the hypothesis that physical characteristics of UPFs influence physiological satiety independently of nutrient composition.

Several mechanisms have been proposed to explain these observations. Many UPFs possess reduced structural integrity because industrial processing disrupts the natural food matrix. Consequently, these foods require less chewing, are consumed more rapidly, and pass through the gastrointestinal tract differently from minimally processed foods. Faster eating rates reduce oro-sensory exposure, delaying activation of satiety signals and increasing meal size (15). Combined with high energy density, these characteristics create conditions that favour chronic positive energy balance.

The implications of these findings are particularly relevant to Qatar. Rapid urbanisation has substantially altered food availability, with energy-dense fast foods, convenience meals, sugar-sweetened beverages and packaged snacks now widely accessible throughout the country. Children and adolescents are increasingly exposed to these products through schools, shopping malls, food delivery platforms and digital marketing. Consequently, habitual consumption of energy-dense UPFs may contribute to excessive caloric intake during critical stages of growth and development.

Overall, evidence supporting the role of energy density in obesity is strong. Randomised controlled feeding trials consistently demonstrate increased energy intake and short-term weight gain during UPF consumption (13, 11), while systematic reviews report similar findings across observational studies (13). Nevertheless, intervention studies have generally involved relatively small sample sizes and short follow-up periods, limiting conclusions regarding long-term obesity development. Furthermore, observational studies remain susceptible to residual confounding from physical activity, socioeconomic status and broader dietary behaviours.

## 4 Portion size effects

In addition to high energy density, increasing portion sizes have become an important environmental driver of obesity (15). During recent decades, commercially available portion sizes have increased substantially across many processed and ultra-processed food products, encouraging greater energy intake while simultaneously altering perceptions of what constitutes an appropriate serving.

Evidence indicates that larger portions consistently increase food consumption irrespective of hunger or body weight. Papagiannaki and Kerr identified increasing portion sizes as a key contributor to the modern obesogenic food environment (15). Originally documented in North America and Europe, this trend has subsequently become evident worldwide as multinational food manufacturers and restaurant chains have expanded globally.

Experimental studies provide strong evidence supporting a causal relationship between portion size and energy intake. Rolls et al. demonstrated that increasing portion size significantly increased energy intake among both normal-weight and overweight adults, independent of hunger (16). Similar effects have subsequently been reported across a wide range of foods, including snack products, refined carbohydrate-rich meals, confectionery, popcorn and cakes (16, 17).

Comparable trends have been observed for beverages. Flood et al. demonstrated that increasing beverage portion size resulted in significantly greater caloric intake during meals, while Young and Nestle highlighted the progressive increase in commercially available beverage sizes over recent decades (18, 19). These findings are particularly concerning because sugar-sweetened beverages contribute substantial energy without producing equivalent satiety responses, thereby facilitating excess caloric intake.

Among adolescents, portion size appears particularly influential. Albar et al. reported that larger portion sizes of energy-dense foods, including cakes, biscuits, cream and soft drinks, were associated with higher body mass index among British adolescents (20). Although the cross-sectional design precludes causal inference, the findings suggest that repeated exposure to larger portions during adolescence may contribute to long-term eating behaviours and obesity risk.

Psychological mechanisms also contribute to portion-size effects. Consumers frequently perceive larger servings as offering better economic value, encouraging selection of oversized meals. Repeated exposure to large portions gradually recalibrates perceptions of normal serving size, resulting in greater self-served portions during subsequent meals. Raghoebar et al. demonstrated that participants previously exposed to smaller portions subsequently selected smaller portions themselves, suggesting that portion-size norms remain modifiable (21).

The influence of tableware has also received considerable attention. Hollands et al. concluded that increasing portion size, package size and tableware dimensions generally increases food consumption, whereas reducing these environmental cues may decrease intake (22). In contrast, Robinson et al. found less consistent evidence regarding the independent effects of plate size, suggesting that tableware alone is unlikely to explain overeating (23). These conflicting findings illustrate the complexity of eating behaviour and indicate that portion size probably interacts with multiple behavioural and environmental factors rather than acting independently.

Within Qatar, increasing availability of oversized restaurant meals, buffet dining, food delivery services and value promotions may further reinforce excessive portion consumption. International fast-food chains frequently market larger meal combinations at relatively small additional cost, encouraging consumers to select larger portions. Such practices may be particularly influential among adolescents, whose food choices are strongly shaped by convenience, affordability and marketing.

Evidence supporting the role of portion size in promoting excessive energy intake is robust, particularly from experimental studies demonstrating a direct causal relationship between larger portions and increased food consumption (15–23).

## 5 Effects on satiety and appetite regulation

One of the principal mechanisms through which processed and ultra-processed foods (UPFs) promote excessive energy intake is by disrupting the physiological systems responsible for regulating hunger and satiety. Appetite regulation is a highly integrated process involving mechanical gastric distension, gastrointestinal hormone secretion, nutrient sensing, neural signaling via the gut–brain axis, and central nervous system reward pathways. The structural and nutritional characteristics of UPFs appear to interfere with several of these regulatory processes simultaneously, increasing the likelihood of overconsumption.

### 5.1 Disruption of physiological satiety signaling

The gastrointestinal tract secretes numerous peptide hormones that regulate appetite following food consumption. Among the most extensively studied are peptide YY (PYY) and glucagon-like peptide-1 (GLP-1), both of which are released from enteroendocrine cells following nutrient ingestion and act through the gut–brain axis to promote satiety (24). These hormones slow gastric emptying, reduce appetite and contribute to meal termination.

Importantly, hormonal release depends not only on nutrient composition but also on the physical properties of food. Food texture, viscosity, fibre content and chewing duration all influence gastrointestinal hormone secretion (25). Foods requiring prolonged mastication increase oral exposure time and gastric distension, thereby enhancing secretion of satiety hormones. In contrast, many UPFs are intentionally engineered to possess soft textures that require minimal chewing, allowing rapid consumption with reduced oro-sensory stimulation.

Argyrakopoulou et al. demonstrated that participants consuming an identical meal over 30 min exhibited significantly higher circulating concentrations of PYY and GLP-1 than participants consuming the same meal within five minutes (26). Slower eating was also associated with lower subjective hunger ratings. These findings provide strong physiological evidence that eating rate directly influences hormonal regulation of appetite.

UPFs frequently possess characteristics that encourage rapid ingestion, including soft textures, low fibre content and high palatability (3, 25). Consequently, habitual consumption of these foods may attenuate normal satiety responses, resulting in larger meal sizes and increased total daily energy intake. Although most mechanistic studies have been conducted under controlled laboratory conditions, their findings are remarkably consistent and biologically plausible.

High dietary fibre further contributes to satiety by delaying gastric emptying, increasing gastric distension and prolonging nutrient absorption (4). However, diets dominated by UPFs consistently contain substantially lower fibre than diets based on minimally processed foods (3, 4). Reduced fibre intake therefore compounds the diminished hormonal response associated with rapid eating, further promoting excessive food consumption.

### 5.2 Rapid gastric emptying and absorption

The physical structure and composition of processed and fast foods promote rapid gastric emptying and nutrient absorption, leading to brief satiety duration and earlier return of hunger compared to whole foods (27). The homogenized, pre-processed nature of many processed foods requires minimal gastric processing, the digestive accelerating transit through system.

Fast food preparation methods often create foods with minimal structural integrity that break down rapidly during digestion. Deep frying creates crispy exteriors that quickly dissolve in gastric acid, while the soft interiors of burgers, processed chicken, and similar items require minimal mechanical breakdown. This rapid breakdown accelerates gastric emptying and reduces the duration of mechanical satiety signals (27).

Research comparing identical macronutrient compositions in whole versus processed forms consistently demonstrates faster gastric emptying for processed versions. Studies by Flood-Obbagy and Rolls (2009) found that whole apples required significantly longer gastric emptying times compared to applesauce or apple juice, despite identical caloric and macronutrient content (18). Similar patterns have been observed comparing whole grains to refined grain products, fresh vegetables to processed vegetable products, and minimally processed meats to processed meat products (12).

The rapid absorption characteristics of processed foods also contribute to shorter satiety duration. The pre-processing of carbohydrates, proteins, and fats in manufactured foods accelerates their digestion and absorption in the small intestine, leading to rapid nutrient appearance in the bloodstream followed by equally rapid clearance. This pattern contrasts with the slower, more sustained absorption patterns seen with whole foods, which provide longer-lasting satiety effects (28).

Although these mechanisms have been demonstrated primarily in controlled feeding studies, they provide a compelling biological explanation for the increased energy intake consistently observed in randomised intervention trials involving UPFs. Nevertheless, longer-term studies are required to determine whether these short-term physiological responses translate into sustained weight gain under free-living conditions.

### 5.3 Glycaemic response and insulin sensitivity

UPFs have also been implicated in alterations of postprandial glycaemic control.

Capra et al. compared diets high and low in UPFs and found no significant differences in insulin sensitivity as measured using HOMA-IR and the Matsuda Index (29). However, participants consuming the UPF-rich diet demonstrated significantly greater glycaemic variability following meals.

Although these findings suggest that short-term insulin sensitivity may remain unchanged, greater glucose fluctuations may shorten satiety duration and increase the frequency of hunger, thereby encouraging additional snacking throughout the day.

Emerging evidence also suggests that food additives commonly present within UPFs may indirectly influence glucose homeostasis through alterations of the gut microbiota (30). Experimental studies have reported that emulsifiers such as carboxymethylcellulose and polysorbate-80 may impair intestinal barrier function and promote metabolic inflammation, although much of this evidence currently derives from animal models.

Furthermore, high UPF consumption has been associated with increased prevalence of metabolic dysfunction-associated steatotic liver disease (MASLD) and non-alcoholic fatty liver disease (NAFLD) (31, 32). Hepatic lipid accumulation contributes to insulin resistance through activation of intracellular signalling pathways involving protein kinase C epsilon (PKCε), thereby impairing insulin receptor function (33).

Although mechanistic evidence linking UPFs with impaired glucose regulation continues to expand, direct causal evidence remains limited. Many studies are cross-sectional and cannot distinguish whether metabolic dysfunction results from UPF consumption itself or from obesity and associated lifestyle behaviours. Consequently, interpretation should remain cautious until longer-term intervention studies become available.

Overall, evidence linking UPFs with impaired appetite regulation is moderately strong. Randomised feeding studies consistently demonstrate reduced satiety, faster eating rates and greater energy intake during UPF consumption (3, 11), while mechanistic investigations support plausible hormonal, gastrointestinal and metabolic pathways (12, 18, 24–33). Nevertheless, most intervention studies involve relatively short follow-up periods and controlled laboratory settings.

## 6 Nutritional displacement

Beyond promoting excessive caloric intake, processed and ultra-processed foods contribute to obesity by displacing nutrient-dense whole foods from the diet. This mechanism reflects changes in overall dietary quality rather than simply increases in energy intake and represents an important consequence of the ongoing nutrition transition observed worldwide.

Traditionally balanced dietary patterns rich in fruits, vegetables, whole grains, legumes, lean protein and dairy products provide dietary fibre, essential vitamins, minerals and numerous bioactive compounds that support growth, metabolic health and appetite regulation (34, 35). In contrast, UPFs typically provide large quantities of refined carbohydrates, saturated fats, added sugars and sodium while containing substantially lower concentrations of micronutrients and dietary fibre (3, 4).

Consequently, increasing consumption of UPFs frequently replaces healthier foods rather than supplementing them. This phenomenon has been consistently observed across both children and adults and is associated with poorer overall dietary quality, reduced micronutrient intake and greater obesity risk.

Among children and adolescents, nutritional displacement may have particularly important consequences because nutritional requirements are high during growth and development (35). Diets dominated by packaged snacks, confectionery, fast foods and sugar-sweetened beverages frequently result in lower consumption of fruits, vegetables and whole grains.

Sugar-sweetened beverages illustrate this displacement particularly well. Malik et al. demonstrated that regular consumption of sugar-sweetened beverages is associated with increased weight gain while simultaneously replacing nutritionally valuable drinks such as milk and water (36). Consequently, excessive intake not only increases caloric consumption but also reduces intake of calcium, protein and other essential nutrients.

Environmental influences further reinforce this dietary transition. Processed foods are heavily marketed to children through colourful packaging, digital media, celebrity endorsements and product placement (6, 37). Their convenience, relatively low preparation requirements and widespread availability often make them the default dietary choice for families experiencing increasingly busy lifestyles.

These environmental changes are especially relevant within Qatar. Rapid urbanisation, increasing household income and widespread availability of international food retailers have substantially altered dietary habits among young people. As mentioned in section 1.1, traditional Qatari diets based on foods such as fish, dates, and legumes are being increasingly replaced by commercially prepared fast foods and packaged snacks (7, 8). Food delivery applications and international restaurant chains have further increased access to energy-dense convenience foods, particularly among adolescents.

Importantly, nutritional displacement influences obesity through several interconnected pathways. Diets low in fibre and protein but rich in refined carbohydrates generally produce weaker satiety responses, encouraging more frequent eating (38). Simultaneously, chronic deficiencies in vitamins, minerals and other bioactive nutrients may contribute to metabolic dysfunction and increased susceptibility to diet-related non-communicable diseases (NCDs) (39).

The cumulative effect is therefore not merely excessive calorie consumption but progressive deterioration in overall dietary quality, which may increase lifelong risks of obesity, cardiovascular disease, type 2 diabetes and other chronic conditions.

Evidence supporting nutritional displacement is strong and highly consistent across observational studies and systematic reviews. Individuals consuming higher proportions of UPFs generally exhibit poorer diet quality and lower intakes of essential nutrients (3, 34–40). However, most evidence remains observational and relies on self-reported dietary assessment, introducing potential recall bias and measurement error. Furthermore, dietary quality may be influenced by broader socioeconomic factors including food affordability, education and household food environments.

## 7 Neurobiological consequences of UPF consumption

In addition to disrupting physiological appetite regulation, increasing evidence suggests that habitual consumption of ultra-processed foods (UPFs) may alter central nervous system pathways involved in food reward, motivation and eating behaviour. Unlike minimally processed foods, many UPFs are specifically engineered to maximise palatability through combinations of refined carbohydrates, added sugars, fats, salt and flavour enhancers. These formulations strongly stimulate neural reward circuits, potentially reinforcing repeated consumption beyond physiological energy requirements (41).

The neurobiology of food intake involves continuous interaction between homeostatic mechanisms regulating energy balance and hedonic pathways that govern food reward. While homeostatic regulation primarily involves hypothalamic integration of peripheral hormonal signals such as leptin, insulin, glucagon-like peptide-1 (GLP-1) and peptide YY (PYY), hedonic eating is largely mediated through dopaminergic pathways within the mesocorticolimbic reward system. Increasing evidence indicates that UPFs disproportionately activate these reward circuits, increasing food craving and encouraging repeated consumption despite adequate energy stores (41, 42).

### 7.1 Reward pathways and addictive eating behaviours

The mesocorticolimbic reward system comprises interconnected regions including the ventral tegmental area, nucleus accumbens, ventral pallidum and hypothalamus, which collectively regulate motivation, pleasure and reinforcement associated with food intake (41). Consumption of highly palatable foods stimulates dopamine release within these regions, producing rewarding sensations that reinforce eating behaviour.

Unlike naturally occurring foods, UPFs frequently combine refined sugars, fats and sodium in concentrations rarely encountered in whole-food diets. This combination appears to produce disproportionately strong activation of reward pathways, leading some investigators to suggest that habitual UPF consumption shares behavioural characteristics with addictive disorders. Although the concept of “food addiction” remains controversial, growing evidence indicates that repeated exposure to highly palatable foods may promote compulsive eating behaviours characterised by loss of control, persistent craving and continued consumption despite adverse health consequences (41).

Human neuroimaging studies increasingly support these observations. Morales and Berridge described distinct neurobiological mechanisms underlying food “liking” (hedonic pleasure) and “wanting” (motivational drive), demonstrating that repeated stimulation of reward circuitry may strengthen motivational responses independently of physiological hunger (42). Consequently, individuals may continue consuming highly palatable foods even when energy requirements have been met, contributing to chronic positive energy balance.

These findings are particularly relevant during childhood and adolescence. Neural reward systems continue to mature throughout adolescence, potentially increasing susceptibility to highly rewarding food stimuli during this developmental period. Combined with intensive marketing of UPFs to young people, repeated activation of reward pathways may establish lifelong dietary preferences that are difficult to modify in adulthood.

### 7.2 Structural brain changes associated with UPF consumption

Recent neuroimaging studies suggest that habitual UPF consumption may also be associated with structural alterations in brain regions involved in appetite regulation and reward processing.

Morys et al. demonstrated that higher UPF consumption was associated with altered white matter microstructure within feeding-related brain regions, independent of overall adiposity (43). Specifically, increased mean diffusivity and reduced fractional anisotropy within the right nucleus accumbens suggested reduced structural integrity of neural pathways involved in reward regulation.

Although these findings remain observational, they provide preliminary evidence that dietary patterns may influence brain structure independently of obesity itself. Importantly, however, the direction of causality remains uncertain. It is currently unclear whether altered brain structure predisposes individuals to consume greater quantities of UPFs or whether long-term UPF consumption contributes directly to neurobiological changes. Longitudinal neuroimaging studies will therefore be essential to clarify these relationships.

Beyond reward circuitry, obesity itself has been associated with widespread alterations in brain structure. Increased visceral adiposity has been linked to reductions in grey matter volume and greater white matter lesion burden (44). These findings suggest that chronic consumption of energy-dense diets may contribute not only to obesity but also to progressive deterioration of neurological health.

### 7.3 Gut-brain communication and GABAergic signaling

The relationship between food intake and brain function is mediated through continuous communication between the gastrointestinal tract and central nervous system, commonly referred to as the gut-brain axis. Neural signalling via the vagus nerve integrates peripheral sensory information with hypothalamic centres responsible for appetite regulation.

Recent experimental studies have identified gamma-aminobutyric acid (GABA) as an important neurotransmitter involved in regulating feeding behaviour. Martinez de Morentin et al. demonstrated that inhibition of GABAergic neurons projecting from the dorsal vagal complex to the arcuate nucleus stimulated feeding behaviour in satiated mice, suggesting that these pathways normally suppress appetite (45).

Similarly, Castro et al. highlighted the interaction between GABAergic signalling and mesocorticolimbic reward pathways involved in motivated feeding. Together, these findings suggest that repeated consumption of highly palatable foods may influence both homeostatic and hedonic regulation of appetite.

However, it is important to recognise that much of the mechanistic evidence regarding GABAergic signalling currently derives from animal models. While these studies provide valuable insight into biological pathways, their direct applicability to human eating behaviour remains uncertain and requires confirmation in clinical studies.

### 7.4 Neuroinflammation and metabolic dysfunction

Emerging evidence also suggests that chronic consumption of diets rich in saturated fat may promote neuroinflammation, providing an additional mechanism linking UPFs with obesity.

Microglia are the resident immune cells of the central nervous system and play essential roles in maintaining neuronal homeostasis and responding to injury (46). Experimental studies demonstrate that diets rich in saturated fat stimulate microglial activation, leading to increased production of pro-inflammatory cytokines within the hypothalamus.

Valdearcos et al. demonstrated that mice consuming high-fat diets exhibited significantly greater hypothalamic inflammation, larger activated microglia and impaired neuronal function compared with animals consuming standard laboratory diets (47). These inflammatory changes were associated with altered appetite regulation and weight gain.

Although these findings are biologically compelling, most evidence currently derives from animal studies. Human studies investigating neuroinflammation following habitual UPF consumption remain limited, and it is therefore premature to conclude that identical mechanisms operate in children and adolescents consuming Westernised diets. Nevertheless, chronic low-grade inflammation is recognised as an important feature of obesity, suggesting that inflammatory pathways may represent an important area for future investigation.

### 7.5 Relevance to Qatar

The potential neurobiological effects of UPFs are particularly relevant within Qatar because children and adolescents are increasingly exposed to highly palatable foods through expanding fast-food markets, digital advertising and food delivery applications (7–9). These environmental changes coincide with developmental periods during which lifelong eating habits and food preferences are established. Consequently, repeated activation of neural reward pathways may reinforce preference for energy-dense foods while simultaneously reducing acceptance of traditional diets rich in minimally processed foods (see Section 1.1).

Understanding these neurobiological mechanisms therefore extends beyond explaining obesity alone. It also highlights why educational interventions that rely solely on individual willpower or knowledge may have limited effectiveness when food environments continuously promote highly rewarding dietary choices. Public health strategies should therefore seek to modify food environments in addition to promoting individual behaviour change.

Overall, evidence supporting neurobiological mechanisms linking UPFs with obesity is emerging but remains less robust than evidence supporting energy density, portion size and appetite regulation (Table 2). Human neuroimaging studies increasingly demonstrate associations between UPF consumption and altered brain structure (44), while experimental studies provide plausible mechanistic explanations involving dopamine signalling, GABAergic pathways and neuroinflammation (43, 45, 47). However, many mechanistic investigations rely on animal models or cross-sectional human studies, limiting causal inference. Future longitudinal neuroimaging studies and controlled dietary interventions conducted in children and adolescents are required to determine whether these observed neurobiological alterations precede obesity or arise as a consequence of excess adiposity.

**Table 2:** Biological and behavioural mechanisms linking ultra-processed foods with childhood obesity.

Mechanism	Underlying biological process	Potential consequence	Key references
Increased energy density	Higher calories per gram with reduced fibre and water	Passive overconsumption	(4, 11, 13)
Larger portion sizes	Environmental cues increase meal size	Increased total daily energy intake	(15–23)
Appetite and satiety dysregulation	Reduced GLP-1 and PYY responses, faster eating, rapid gastric emptying	Earlier return of hunger	(12, 18, 24–33)
Nutritional displacement	Replacement of fruits, vegetables and whole grains by UPFs	Reduced diet quality and micronutrient intake	(34–40, 42)
Reward-driven eating	Dopaminergic activation of mesocorticolimbic pathways	Increased food craving and compulsive eating	(41–45)
Neuroinflammation	Microglial activation and hypothalamic inflammation	Altered appetite regulation and metabolic dysfunction	(46, 47)

## 8 The shift from the traditional Qatari diet

The present review synthesised current evidence describing five interconnected biological and behavioural mechanisms through which processed and ultra-processed foods (UPFs) may contribute to obesity among children and adolescents. Collectively, the available evidence suggests that UPFs influence energy balance through multiple complementary pathways rather than through excess calorie consumption alone. These mechanisms include increased energy density and portion size, impaired appetite and satiety regulation, nutritional displacement of whole foods, activation of neural reward pathways, and emerging neurobiological and metabolic alterations. Although each mechanism has been investigated independently, increasing evidence indicates that they interact synergistically to reinforce excessive energy intake and long-term weight gain.

One of the strongest findings emerging from this review is the consistency of evidence linking UPF consumption with increased energy intake. Randomised controlled feeding studies demonstrate that diets rich in UPFs promote significantly greater caloric intake and short-term weight gain even when matched for macronutrient composition (3, 11). These findings provide stronger evidence than observational studies because they minimise confounding and establish a temporal relationship between dietary exposure and weight gain. Nevertheless, most intervention studies have been conducted over relatively short periods, typically ranging from one to four weeks. Consequently, while these studies demonstrate biological plausibility, they provide limited information regarding the long-term development of obesity or whether similar effects persist under free-living conditions.

Evidence relating to appetite regulation is also compelling. Controlled laboratory studies consistently demonstrate that food texture, eating rate and fibre content influence secretion of satiety hormones including peptide YY and glucagon-like peptide-1 (24–26). Furthermore, diets rich in UPFs generally reduce oral processing time while accelerating gastric emptying and nutrient absorption (12, 27, 28, 48). Together, these physiological effects provide a plausible explanation for increased meal size and more frequent eating observed during UPF consumption. However, most mechanistic investigations have been undertaken under highly controlled experimental conditions. Eating behaviours in free-living populations are additionally influenced by social, environmental and psychological factors that are difficult to replicate within laboratory settings.

Nutritional displacement emerged as another important mechanism. Rather than simply increasing energy intake, UPFs frequently replace fruits, vegetables, whole grains and other minimally processed foods, thereby reducing dietary quality (34–40). This process may have particularly important consequences during childhood and adolescence when adequate micronutrient intake is essential for growth and development. However, dietary displacement is also influenced by broader environmental determinants including food affordability, marketing, parental behaviours and household food availability. Consequently, interventions targeting UPF consumption should consider overall dietary patterns rather than focusing exclusively on individual food products.

The evidence supporting neurobiological mechanisms remains comparatively less mature. Neuroimaging studies suggest that habitual UPF consumption may influence brain regions involved in reward processing (43), while experimental studies demonstrate biologically plausible pathways involving dopamine signalling, GABAergic neurons and hypothalamic inflammation (41, 45, 47). However, much of this evidence derives from animal models or cross-sectional imaging studies. Whether these neurobiological alterations represent causes or consequences of obesity remains uncertain. Further longitudinal neuroimaging research is therefore required before definitive conclusions can be drawn.

### 8.1 Strengths and limitations of the current evidence

An important observation arising from this review is the variation in methodological quality across the literature. Systematic reviews, meta-analyses and randomised controlled trials provide the strongest evidence supporting associations between UPFs and obesity. However, a substantial proportion of the available literature consists of observational studies, which remain vulnerable to residual confounding, reverse causation and measurement error. Dietary intake is commonly assessed using food-frequency questionnaires or dietary recalls, both of which are susceptible to recall bias and under-reporting, particularly among individuals living with overweight or obesity.

Furthermore, definitions of processed and ultra-processed foods remain inconsistent across studies. Although the NOVA classification system has become widely accepted, differences in food categorisation and dietary assessment methods complicate direct comparison between investigations. Standardisation of dietary assessment methodologies would substantially strengthen future evidence synthesis.

### 8.2 Relevance to Qatar

Although the biological mechanisms described in this review are likely to operate universally, their public health implications are particularly relevant to Qatar. The country has experienced one of the most rapid nutrition transitions globally, characterised by increased household income, urbanisation, widespread availability of imported foods and changing lifestyle behaviours (7–9). These changes have coincided with rising childhood obesity and increasing dependence on commercially prepared foods (see Section 1.1).

Unlike many Western countries, where dietary transitions occurred gradually over several decades, Qatar has experienced these changes within a relatively short timeframe. Consequently, multiple environmental determinants—including international fast-food chains, food delivery platforms, reduced physical activity, increasing screen time and aggressive food marketing—have emerged simultaneously. This convergence may amplify the biological mechanisms described throughout this review and contribute to the exceptionally high prevalence of childhood obesity observed in the country.

Additionally, the limited available research in the Middle East and GCC warrants future research to focus specifically on these populations through longitudinal cohort studies, providing further insight into the relationship between factors such as UPF consumption, energy density, food texture, portion size, and family food practices and childhood obesity. This would prompt evidence-based policy change and government efforts that would be most effective in the Qatari cultural landscape.

## 9 Implications for public health and policy

The findings of this review suggest that reducing childhood obesity requires interventions extending beyond individual behaviour change. Because processed and ultra-processed foods influence obesity through multiple interconnected mechanisms, effective prevention strategies should combine policy initiatives, environmental modifications and educational interventions.

### 9.1 Public health policy

Policy interventions should prioritise improving food environments for children and adolescents. Potential strategies include restricting marketing of UPFs to children, strengthening nutrition standards within schools, improving front-of-pack food labelling, promoting healthier food procurement policies and encouraging reformulation of commercially available products. Fiscal measures such as taxation of sugar-sweetened beverages have demonstrated promising results internationally and may complement existing public health initiatives within Qatar.

### 9.2 Clinical practice

Healthcare professionals should routinely assess dietary quality in addition to body weight during paediatric consultations. Early identification of high UPF consumption may facilitate timely dietary counselling before obesity becomes established. Multidisciplinary approaches involving paediatricians, dietitians, psychologists and school health professionals are likely to be most effective for supporting long-term behaviour change.

### 9.3 Nutrition education

Educational interventions remain essential but should extend beyond traditional nutrition advice. Improving food literacy among children, parents and teachers may enhance understanding of food processing, portion size, food labels and healthy meal preparation. Reintroducing elements of traditional Qatari dietary practices may also provide culturally appropriate strategies for improving dietary quality while preserving national food heritage.

## 10 Limitations and future research directions

Several limitations should be considered when interpreting the findings of this review. First, although a structured narrative methodology was employed, the review was not designed as a systematic review and therefore did not include formal risk-of-bias assessment or quantitative meta-analysis. Nevertheless, transparent search methods and predefined eligibility criteria were used to improve methodological rigour.

Second, much of the available evidence originates from Europe and North America. Studies conducted within Qatar and the wider GCC remain comparatively limited. Although international studies provide valuable mechanistic insight into the interplay between UPFs and obesity, substantial differences in dietary practices, family structures, cultural norms and food environments between regions may reduce confidence in the direct applicability of some findings to local populations.

Third, considerable heterogeneity exists across studies regarding dietary assessment methods, definitions of UPFs, participant characteristics and reported outcome measures. This variability limited direct comparison between studies and prevented quantitative synthesis.

Finally, several proposed mechanisms, particularly neurobiological alterations and gut-brain interactions, remain supported primarily by experimental or animal research. Further human intervention studies are required before definitive causal conclusions can be established.

Future research should focus on strengthening evidence specific to Qatar and the wider Gulf region. Priority areas include:

- Large prospective cohort studies examining habitual UPF consumption during childhood;
- Long-term dietary intervention trials evaluating reduction of UPF intake;
- School-based nutrition interventions;
- Evaluation of national food policies and marketing regulations;
- Investigation of gut microbiome responses to dietary transition;
- Longitudinal neuroimaging studies examining food reward pathways;
- Improved dietary assessment methods using objective biomarkers;
- Evaluation of culturally tailored interventions promoting traditional Qatari dietary practices.

Addressing these priorities will provide stronger evidence to guide future public health policy and obesity prevention strategies within Qatar.

## 11 Conclusion

Childhood and adolescent obesity continues to represent one of the most significant public health challenges facing Qatar. Rapid economic development, urbanisation, and changes in food environments have resulted in a substantial nutrition transition, characterised by increasing consumption of processed and ultra-processed foods (UPFs) and declining adherence to traditional dietary patterns. The evidence synthesised in this review suggests that UPFs contribute to obesity through multiple interconnected biological and behavioural mechanisms rather than through excessive calorie intake alone.

**Figure 1:**
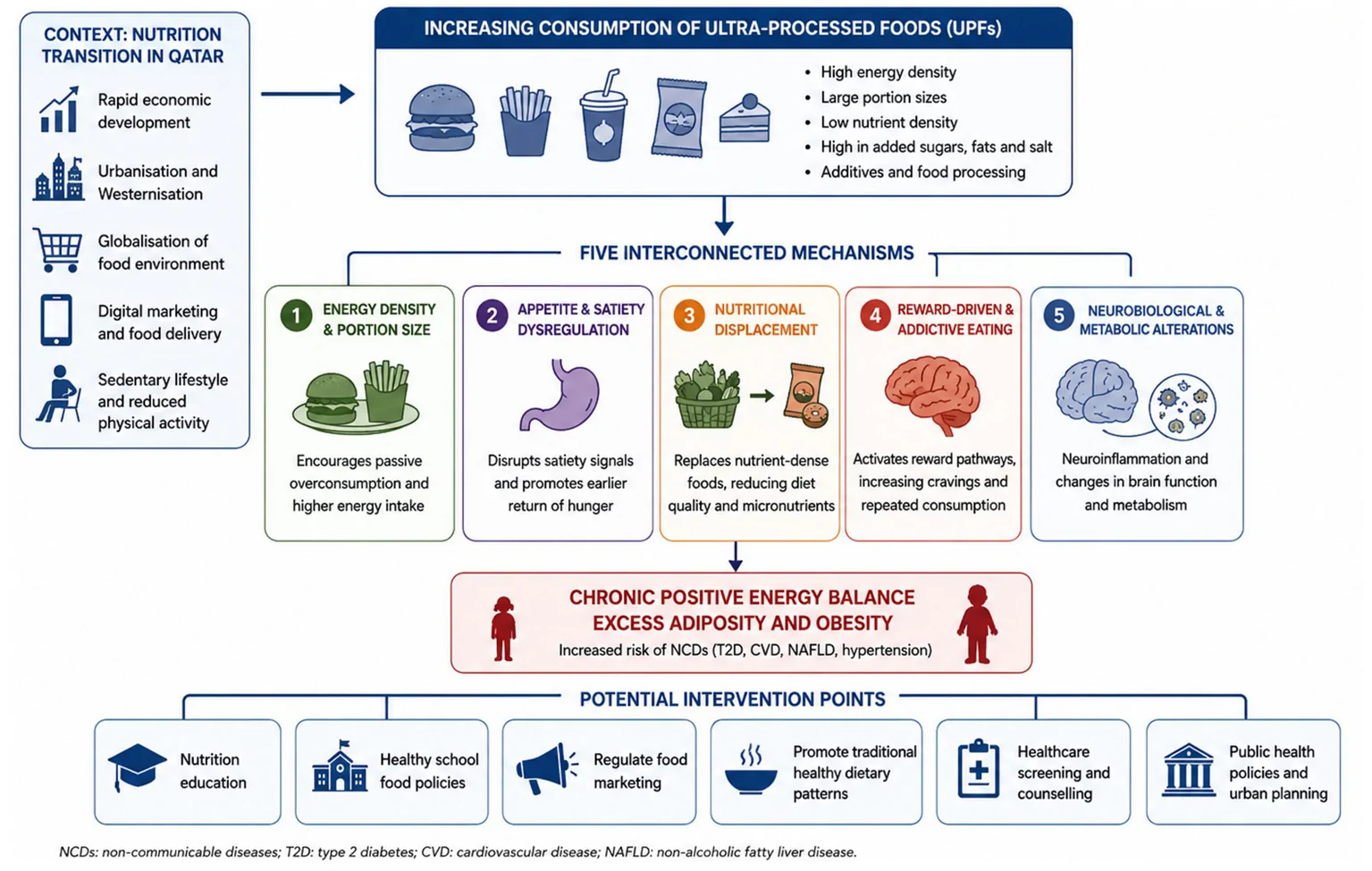
Proposed mechanisms linking ultra-processed food consumption to obesity among children and adolescents in Qatar.

This review identified five principal mechanisms through which UPFs may promote obesity in children and adolescents (Figure 1). First, their high energy density and increasingly large portion sizes encourage passive overconsumption and positive energy balance. Second, their physical and nutritional characteristics disrupt normal appetite and satiety regulation by altering eating rate, gastric emptying and gut hormone secretion. Third, frequent consumption of UPFs displaces nutrient-dense whole foods, reducing dietary quality and increasing the risk of micronutrient inadequacy. Fourth, highly palatable formulations stimulate reward pathways within the brain, reinforcing repeated consumption through hedonic eating behaviours. Finally, emerging evidence suggests that habitual UPF consumption may contribute to structural and functional neurobiological alterations, although further human studies are required to confirm these mechanisms.

Collectively, these pathways demonstrate that obesity is not simply the result of individual food choices or excessive willpower but reflects a complex interaction between biological susceptibility and increasingly obesogenic food environments. This perspective is particularly important in Qatar, where rapid changes in food availability, marketing, urbanisation and lifestyle have occurred within a relatively short period and may amplify the effects of UPFs on childhood obesity.

One of the principal findings of this review is that the overall evidence supporting an association between UPFs and obesity is substantial and continues to strengthen. Randomised controlled feeding studies provide convincing evidence that diets rich in UPFs increase daily energy intake and body weight, while systematic reviews and prospective cohort studies consistently report positive associations between UPF consumption and obesity across diverse populations (3, 11, 13). Nevertheless, important limitations remain. Much of the available literature has been conducted in Europe and North America, and relatively few studies have examined these relationships within Middle Eastern populations. Furthermore, evidence relating to neurobiological mechanisms and gut–brain communication remains at an early stage, with many mechanistic insights derived from experimental or animal studies.

These findings highlight the need for obesity prevention strategies that extend beyond individual dietary advice. Reducing childhood obesity in Qatar will require coordinated action across multiple sectors, including healthcare, education, food policy and urban planning. Interventions should focus on improving the nutritional quality of school food environments, reducing children’s exposure to marketing of ultra-processed foods, encouraging consumption of minimally processed traditional foods, improving nutrition literacy among families, and supporting healthier food choices through national policy initiatives.

Future research should prioritise longitudinal studies conducted within Qatar to better understand dietary behaviours throughout childhood and adolescence, evaluate culturally appropriate intervention strategies, and investigate the long-term biological consequences of UPF consumption (Table 3). Particular emphasis should be placed on prospective cohort studies, school-based intervention trials, and mechanistic research examining the interaction between diet, the gut microbiome, neurobiology and metabolic health. Strengthening the regional evidence base will enable public health recommendations to be tailored more effectively to the cultural, environmental and socioeconomic context of Qatar and the wider Gulf region.

**Table 3:** Research priorities for Qatar.

Priority area	Rationale	Potential impact
Longitudinal cohort studies	Limited prospective evidence from Qatar	Establish causal relationships between UPFs and obesity
School-based nutrition interventions	Schools represent a key setting for prevention	Improve dietary quality during childhood
Evaluation of food policy	Limited evidence regarding policy effectiveness in Qatar	Inform national obesity prevention strategies
Food marketing research	High exposure of children to digital food marketing	Support regulatory policies
Gut microbiome studies	Emerging mechanistic pathway linking diet and obesity	Identify novel therapeutic targets
Neuroimaging research	Limited understanding of brain adaptations to UPFs	Improve understanding of reward-driven eating
Traditional dietary interventions	Declining adherence to traditional diets	Develop culturally appropriate prevention programmes
Objective dietary assessment	Reliance on self-reported food intake	Improve accuracy of future research

In conclusion, addressing the obesity epidemic among young people in Qatar requires recognition that processed and ultra-processed foods influence health through numerous interacting biological, behavioural and environmental mechanisms. A comprehensive understanding of these pathways provides an essential foundation for developing evidence-based public health strategies capable of reducing childhood obesity and improving long-term health outcomes in future generations.
